# The associations between wearable device feedback perception and athletes’ training load management behavior: the chain mediating role of physiological data literacy and recovery self-regulation

**DOI:** 10.3389/fpsyg.2026.1910446

**Published:** 2026-09-30

**Authors:** Yufeng Han, Junli Fan

**Affiliations:** 1 Department of Physical Education, Jeonbuk National University, Jeonju, Republic of Korea; 2 School of Physical Education, Guangzhou University, Guangzhou, China

**Keywords:** physiological data literacy (PDL), recovery self-regulation (RSR), training load management behavior (TLM), wearable device feedback perception (WFP), wearable technology

## Abstract

**Introduction:**

Despite the rapid proliferation of wearable devices in sport, athletes vary considerably in their ability to interpret device-generated physiological feedback, and the psychological pathways linking feedback perception to training load management remain insufficiently understood. This study introduces physiological data literacy (PDL) as a content-specific form of data literacy and examines whether PDL and recovery self-regulation (RSR) sequentially mediate the association between wearable device feedback perception (WFP) and training load management behavior (TLM).

**Methods:**

Using a cross-sectional design, 357 active athletes (189 males and 168 females; mean age = 22.76 years, SD = 3.68) completed the wearable device feedback perception scale, physiological data literacy scale, recovery self-regulation scale, and training load management behavior scale. Path analysis with bias-corrected bootstrapping (5,000 resamples) was conducted.

**Results:**

WFP showed a positive total association with TLM (*β* = 0.511). Significant specific indirect associations were observed through PDL (standardized indirect effect = 0.083, 95% CI [0.032, 0.136]) and RSR (standardized indirect effect = 0.085, 95% CI [0.048, 0.128]). A significant sequential indirect association was also observed for the theoretically specified WFP → PDL → RSR → TLM ordering (indirect effect = 0.065, 95% CI [0.040, 0.098]). The direct WFP–TLM association remained significant after inclusion of both mediators. However, the reverse sequential model yielded identical AIC and BIC values.

**Discussion:**

The findings are statistically compatible with a theoretically motivated sequential framework linking WFP, PDL, RSR, and TLM, but the cross-sectional design does not establish temporal precedence or causal direction. PDL represents a conceptually distinct competency that, together with RSR, warrants further examination in longitudinal and intervention research on wearable-supported training.

## Highlights

- Wearable device feedback perception is positively associated with athletes’ self-reported training load management behavior.
- The data were consistent with the proposed WFP → PDL → RSR → TLM pathway, but the cross-sectional design does not establish temporal precedence among these variables.
- The cross-sectional design precludes causal inference; longitudinal and experimental studies are needed.
- All measures were self-reported; future research should incorporate objective training data and coach evaluations.

## 1 Introduction

In contemporary competitive sport, training load management has emerged as a central concern at the intersection of sport science, sports medicine, and sport psychology. Although training load monitoring has been extensively studied, the field lacks a theoretically delineated construct that captures athletes’ ability to interpret the physiological signals embedded in wearable-device feedback. Existing constructs—including health literacy (Nutbeam, 2008), digital and digital-health literacy (Ban et al., 2024), technology-related literacy and acceptance (Venkatesh et al., 2003), and self-monitoring (Carver and Scheier, 1998)—each illuminate different aspects of health information, technology use, or self-regulatory behavior. However, none specifically captures athletes’ capacity to interpret and apply physiological data generated by wearable devices within sport-specific training and recovery decisions. Physiological data literacy (PDL) is introduced to address this more specific conceptual gap.

In recent years, the rapid advancement of wearable technology—including global positioning systems (GPS), inertial measurement units (IMUs), heart-rate monitors, sleep-tracking devices, and multimodal physiological sensors—has substantially transformed athlete monitoring and sports-medicine practice (Düking et al., 2016; Seshadri et al., 2019; Crang et al., 2021; Olsen et al., 2025). Compared to traditional periodic testing, wearable devices enable continuous, real-time, and contextually rich data collection in authentic training environments, providing coaches and athletes with immediate information regarding exercise volume, fatigue, recovery, and potential risk. A systematic review by Crang et al. (2021) demonstrated that wearable microtechnology has been extensively applied in team sports for quantifying movement patterns and external load, driving a paradigm shift in training monitoring from experience-based judgment toward evidence-informed decision-making. The International Olympic Committee consensus statement similarly emphasized that systematic load monitoring is foundational to athlete health protection (Soligard et al., 2016). Meanwhile, the validity and reliability of wearable devices in resistance training monitoring have garnered sustained attention, suggesting that the field is transitioning from a focus on “whether data can be measured” to “how data should be interpreted and utilized with high quality” (Weakley et al., 2021).

Recent advances in artificial intelligence and machine-learning-assisted monitoring systems have expanded the capacity to integrate multidimensional athlete data for performance and health-related decision support (Munoz-Macho et al., 2024; Alkasasbeh et al., 2026). However, increasingly complex algorithm-generated feedback may also place greater demands on users’ ability to interpret physiological information accurately, particularly when machine-learning models provide outputs that are not readily transparent or explainable (Abdelaal et al., 2024). This interpretability challenge further highlights the relevance of physiological data literacy in wearable-supported sport.

Nevertheless, the mere deployment of wearable devices does not automatically translate into scientifically informed training behaviors. Extant research has predominantly concentrated on device accuracy, algorithmic performance, and indicator validity, while relatively neglecting how athletes perceive, comprehend, and utilize device feedback. Recent evidence also indicates that although wearable GPS and accelerometer data are widely used to inform training prescription, considerable variation exists in practitioners’ use and understanding of specific wearable-derived metrics (Dawson et al., 2024). This implementation gap suggests that access to monitoring data does not necessarily ensure accurate interpretation or effective application in training decisions. Training load management is fundamentally a behavioral regulation process that involves athletes’ awareness of their bodily states, interpretation of feedback information, evaluation of training-related risks, and execution of recovery strategies. If athletes perceive device feedback as lacking credibility, being difficult to understand, or inconsistent with their subjective experience, even objectively valid data may be disregarded, misinterpreted, or passively relied upon. The Technology Acceptance Model (TAM) posits that individuals’ perceived usefulness and perceived ease of use of a given technology shape their attitudes toward and behavioral adoption of that technology (Davis, 1989). Accordingly, within competitive training contexts, wearable device feedback perception represents not merely a technological user experience but potentially an important psychological correlate of training load management behavior.

From a sport psychopathology perspective, wearable device feedback carries a dual-edged nature. On the one hand, real-time physiological feedback can improve athletes’ bodily awareness, assisting them in recognizing accumulated fatigue, insufficient recovery, and abnormal load, thereby supporting more proactive training adjustments. On the other hand, excessive attention to data fluctuations may induce anxiety, compulsive monitoring, and catastrophic interpretations of bodily states. Particularly within high-performance sport environments—where athletes are subject to chronic performance pressure, injury risk, and selection competition—indicators such as declining sleep scores, recovery indices, or heart rate variability may be interpreted as a “loss of control” or a “performance threat,” thereby triggering psychological distress and irrational training decisions. The consensus statement on overtraining syndrome notes that maladaptive training responses often co-occur with excessive load, inadequate recovery, and psychological symptoms; athletes who fail to accurately identify and regulate fatigue-recovery states may progress from functional overreaching to non-functional overreaching or even overtraining (Meeusen et al., 2013). Previous research has linked training and competition workload with injury risk, while emphasizing that this relationship is complex and context dependent (Drew and Finch, 2016; Windt and Gabbett, 2017; Ekstrand et al., 2011).

Thus, the association between wearable device feedback perception and training load management behavior is unlikely to occur through a direct pathway alone; rather, it likely requires cognitive processing and self-regulatory mechanisms. First, physiological data literacy may serve as a key cognitive mediator. Data literacy is generally defined as the capacity to comprehend, interpret, evaluate, and apply data in decision-making. Within the sport training context, physiological data literacy can be conceptualized as athletes’ ability to understand, interpret, and utilize physiological data, such as heart rate, heart rate variability (HRV), sleep parameters, fatigue levels, training load zones, and recovery indices. Mandinach and Gummer (2016) emphasized that the core of data literacy lies not in passively receiving data but in responsibly employing multi-source data to support practical decision-making. Transposing this to the competitive sport domain, only when athletes possess foundational physiological data literacy can they distinguish normal fluctuations from risk signals, comprehend the physiological significance underlying various indicators, and translate device feedback into appropriate training and recovery choices.

Second, recovery self-regulation may function as a key behavioral mediator linking cognitive understanding to training load management behavior. Recovery is not a passive process external to training but an integral component of training adaptation. Kellmann et al. (2018), in their sport recovery consensus statement, noted that athletic performance depends on the balance between stress and recovery, and that inadequate recovery undermines sustained high-level performance while elevating health risks. Recovery self-regulation emphasizes athletes’ proactive monitoring of fatigue states, scheduling of sleep, regulation of nutrition, selection of relaxation modalities, control of supplementary training, and execution of recovery plans—all based on bodily and psychological feedback. Drawing on self-regulation theory, individual behavior is governed by goal standards, feedback regarding current states, and discrepancy-reduction processes; when individuals perceive a gap between their current state and their goal state, they initiate corresponding behavioral adjustments (Carver and Scheier, 1998; Zimmerman, 2000). Consequently, if wearable device feedback can be understood and integrated into athletes’ recovery goals, it may facilitate training load management behavior by strengthening recovery self-regulation capacity.

Prior research has also indicated that athlete load monitoring should not rely on a single objective indicator alone. A systematic review by Saw et al. (2016) found that subjective self-report measures hold substantial value in monitoring athlete training responses, and may even outperform commonly used objective measures in certain contexts. This finding underscores that training load management should be understood as a decision-making process that integrates objective data with subjective experience, rather than the mechanical execution of device-derived numerical values. In other words, wearable device feedback can only effectively facilitate training load management behavior when it is perceived by athletes as credible, comprehensible, and actionable, and when it aligns with their bodily experiences and training goals.

Building on the foregoing theoretical and empirical foundations, the present study evaluates a theoretically proposed sequential model involving wearable device feedback perception, physiological data literacy, recovery self-regulation, and training load management behavior. Specifically, we hypothesize that WFP will be positively associated with PDL, that PDL will be positively associated with RSR, and that RSR will be positively associated with TLM, resulting in a significant sequential indirect association for the theoretically specified WFP → PDL → RSR → TLM ordering. This ordering is theoretically motivated but is not assumed to represent established temporal precedence, particularly given the cross-sectional design.

The present study makes three theoretical contributions. First, it extends wearable device research from a focus on technical validity to a focus on cognitive interpretation, by introducing PDL as a content-specific data literacy applicable to physiological indicators in sport. Second, it integrates self-regulation theory by evaluating a theoretically ordered model in which PDL and RSR are positioned as cognitive and self-regulatory components potentially associated with training load management behavior. Third, it provides an empirical benchmark that integrates cognitive and self-regulatory mechanisms in load management, with implications for coaches, sport psychologists, and wearable-device manufacturers.

## 2 Method

### 2.1 Participants and procedure

The present study employed a cross-sectional survey design. Data were collected between January 15 and April 30, 2026, using convenience sampling from five sport universities and four provincial-level sport teams across eastern, northern, and southern China. Inclusion criteria were: (1) age between 18 and 35 years; (2) a minimum of 4 years of systematic sport-specific training at the national second-class athlete level or above; (3) continuous use of wearable devices (e.g., sport watches, heart rate monitors) for monitoring training load and recovery over the preceding 6 months, with a weekly usage frequency of at least three times; and (4) the ability to independently complete an electronic questionnaire via mobile phone. Exclusion criteria were: (1) clinically diagnosed severe sleep disorders or psychiatric conditions; (2) interruption of systematic training for more than 4 weeks due to sports injury within the preceding 3 months; and (3) questionnaire completion time of less than 120 s or clearly patterned responding. Participants were recruited through two complementary channels: (a) sport-university coaches who distributed the Wenjuanxing questionnaire link to eligible athletes, and (b) team administrators at four provincial sport teams who coordinated on-site distribution following a brief standardized briefing. Each questionnaire began with an electronic informed-consent statement, and only participants who clicked “agree” proceeded. Of 423 invitations distributed, 412 athletes opened the questionnaire (cooperation rate = 97.4%); 379 submitted it (response rate = 92.0% of opened questionnaires); 357 were retained after applying exclusion criteria (valid rate = 94.2% of returned questionnaires).

An *a priori* power analysis was conducted using G*Power 3.1 (linear multiple regression, fixed model, R^2^ deviation from zero). Assuming an effect size of f^2^ = 0.15, α = 0.05, statistical power of 0.95, and seven predictors (WFP, PDL, RSR, and four covariates: age, sex, athletic level, and years of training experience), the minimum required sample size was estimated to be 153. The final analytic sample consisted of 357 athletes, exceeding this minimum requirement. Of these participants, 189 were male (52.9%) and 168 were female (47.1%), with a mean age of 22.76 years (SD = 3.68). In terms of athletic level, the sample included 6 international elite athletes (1.7%), 36 national elite athletes (10.1%), 128 first-class athletes (35.9%), and 187 s-class athletes (52.4%). The represented sports included track and field, swimming, cycling, and various ball sports. Participants had an average of 8.35 years of training experience (SD = 3.22) and had used wearable devices for an average of 17.94 months (SD = 10.25). Smartwatches were the primary type of wearable device used (87.4%). The study was approved by the Institutional Review Board (Approval No. 2025–03–011-003), and all participants provided electronic informed consent.

### 2.2 Measures

#### 2.2.1 Wearable device feedback perception scale

Adapted from Davis’s (1989) Technology Acceptance Model and athlete training monitoring research (e.g., Düking et al., 2016; Seshadri et al., 2019), this scale assesses athletes’ subjective perception of feedback information provided by wearable devices. The scale comprises 10 items spanning four dimensions: information comprehensibility (e.g., “The load data displayed by the device is easy to understand”), information timeliness (e.g., “I can immediately see my post-training heart rate recovery”), individual relevance (e.g., “The feedback data reflect my unique personal training responses”), and decision support (e.g., “The feedback information directly helps me plan the next day’s training intensity”). All items center on athletes’ real-time feedback experiences with smartwatches, heart rate monitors, and similar devices in daily training settings. Confirmatory factor analysis (CFA) demonstrated good fit for the four-factor model: χ^2^/df = 2.14, RMSEA = 0.057 (90% CI: 0.040–0.073), CFI = 0.964, TLI = 0.952, SRMR = 0.042. In the present sample, the total scale Cronbach’s α was 0.887, composite reliability (CR) was 0.892, and the average variance extracted (AVE) was 0.675, indicating satisfactory internal consistency and convergent validity. The inter-factor correlations were all smaller than the square root of the AVE (in accordance with the discriminant validity criterion proposed by Fornell and Larcker, 1981), supporting discriminant validity.

#### 2.2.2 Physiological data literacy scale

Developed on the basis of theoretical frameworks of health literacy and data literacy (Nutbeam, 2008; Mandinach and Gummer, 2016), combined with focus group interviews with sport physiology experts, this scale measures athletes’ ability to understand, interpret, and apply physiological data generated by wearable devices. The scale consists of 12 items across three dimensions: data reading (e.g., “I can accurately distinguish between resting heart rate and exercise heart rate”), data interpretation (e.g., “I know that elevated heart rate variability generally indicates good recovery”), and data-based reasoning (e.g., “When blood oxygen saturation remains persistently low, I consider the possibility of overtraining”). All items draw on common physiological indicators encountered in daily life, assessing athletes’ proficiency with real-time heart rate, HRV, blood oxygen saturation, respiratory rate, and related metrics. CFA indicated acceptable fit for the three-factor structure: χ^2^/df = 2.43, RMSEA = 0.063, CFI = 0.951, SRMR = 0.048. The total scale α was 0.901, CR = 0.907, AVE = 0.621, and the square root of the AVE for each factor exceeded the inter-factor correlations, demonstrating good reliability and validity. Additionally, the scale showed a moderate positive correlation with a health literacy questionnaire (r = 0.52, *p* < 0.001), providing further evidence of criterion-related validity.

Scale development note—Physiological Data Literacy Scale. Item generation. The initial pool of 20 items was generated from Mandinach and Gummer's (2016) data literacy framework and three 60–90-min focus-group interviews with five sport-physiology experts (mean experience = 11.4 years), and mapped onto three *a priori* dimensions (data reading, data interpretation, data-based reasoning). Expert panel evaluation. A four-member expert panel (two sport physiology, one sport psychology, one psychometrics) rated each item on a 4-point relevance scale; item-level Content Validity Index (I-CVI) values ranged from 0.75 to 1.00 and Scale-level CVI (S-CVI/Ave) was 0.94. Pilot testing. Eighty-five athletes independent of the main sample completed the preliminary 20-item version; items with corrected item–total correlations below 0.40 or factor loadings below 0.50 were eliminated, leaving the final 12-item scale (pilot Cronbach’s α = 0.89). Criterion-related validity. In the main sample, PDL correlated moderately with a validated health-literacy short form (r = 0.52, *p* < 0.001) and weakly with a generic digital-literacy measure (r = 0.34), supporting conceptual distinctness. Future validation. Independent validation across team-sport vs. individual-sport samples, age strata, and cultural contexts (back-translation with reconciliation) is planned. The Training Load Management Behavior Scale followed an analogous procedure (initial pool n = 14, expert review, pilot n = 85, three-dimension solution), with the additional step of coach-rated criterion validity evidence (r = 0.49, *p* < 0.001).

#### 2.2.3 Recovery self-regulation scale

Grounded in self-regulation theory and sport recovery research (Carver and Scheier, 1982; Kellmann et al., 2018), the Recovery Self-Regulation Scale assesses athletes’ tendencies and strategies for proactively planning, monitoring, and adjusting recovery behaviors. The scale includes 11 items across four dimensions: recovery planning (e.g., “I develop hot-cold contrast recovery protocols in advance based on training load”), internal cue monitoring (e.g., “Upon waking, I immediately assess my level of physical fatigue”), behavioral adjustment (e.g., “When I feel inadequately recovered, I proactively reduce that day’s training intensity”), and environmental optimization (e.g., “I deliberately arrange a sleep environment free from electronic device interference”). Item content focuses on athletes’ proactive management of the physical recovery process within daily training cycles, rather than passive responses. CFA showed good fit for the four-factor model: χ^2^/df = 2.28, RMSEA = 0.060, CFI = 0.957, TLI = 0.946, SRMR = 0.045. The total scale α was 0.872, CR = 0.878, AVE = 0.595. Multi-group CFA supported measurement invariance across male and female athletes, with changes in CFI and RMSEA remaining within commonly recommended criteria (ΔCFI < 0.01, ΔRMSEA < 0.015; Chen, 2007), supporting measurement invariance across gender.

#### 2.2.4 Training load management behavior scale

Developed by integrating load management theory and coach evaluation criteria (Foster et al., 2001; Bourdon et al., 2017), this scale measures athletes’ self-reported behavioral performance in self-regulating training load based on wearable device feedback. The scale comprises 9 items across three dimensions: load monitoring (e.g., “I check the device-recorded training impulse and exercise duration every day”), load communication (e.g., “I discuss the load data from my device with my coach”), and load adjustment (e.g., “When load indicators show a clear upward trend over consecutive days, I proactively reduce training intensity”). Item content comprehensively reflects the full chain through which athletes translate physiological data into specific training management behaviors. CFA demonstrated good fit for the three-factor structure: χ^2^/df = 2.05, RMSEA = 0.054, CFI = 0.968, TLI = 0.959, SRMR = 0.040. In the present sample, the total scale α was 0.864, CR = 0.871, AVE = 0.630. In discriminant validity testing, the square root of the AVE for each dimension exceeded its correlations with other dimensions, and all standardized factor loadings ranged from 0.68 to 0.87 (all *p* < 0.001), indicating satisfactory convergent validity. Moreover, the scale score was significantly and positively correlated with a coach-rated athlete training self-discipline rating scale (r = 0.49, *p* < 0.001), providing external evidence of criterion validity.

### 2.3 Data analysis

Data processing and model testing were conducted using SPSS 27.0 and Mplus 8.3. First, missing value analysis was performed; the rate of random missing data was below 2%, and regression imputation was applied. Kolmogorov–Smirnov tests and assessments of skewness and kurtosis indicated that all observed variables approximated a normal distribution (absolute skewness < 1.0, absolute kurtosis < 1.5), satisfying the assumptions of maximum likelihood estimation. Harman’s single-factor test was used as a descriptive diagnostic of potential common method variance. An unrotated exploratory factor analysis of all items extracted four factors with eigenvalues greater than 1, with the first factor accounting for 43.46% of the total variance. Because all focal constructs were assessed using self-report questionnaires from the same participants at a single measurement occasion, Harman’s test cannot rule out common method variance. Therefore, common method variance cannot be excluded and may have contributed to the magnitude of the observed associations. Regression imputation was selected because Little’s MCAR test indicated that missingness was completely at random (χ^2^ = 312.4, df = 298, *p* = 0.245) and the proportion of missing data per variable was below 2%; under these conditions, regression imputation was considered an acceptable approach for this specific missing-data pattern; nevertheless, unlike multiple imputation, it does not fully propagate imputation uncertainty into standard errors, which is acknowledged as a limitation.

Subsequently, Pearson product–moment correlation coefficients were computed among all core variables, and means and standard deviations were reported to provide a preliminary examination of variable association patterns. Confirmatory factor analysis was employed to test the four-factor measurement model comprising wearable device feedback perception, physiological data literacy, recovery self-regulation, and training load management behavior. The model demonstrated good fit (χ^2^/df = 2.11, RMSEA = 0.056, CFI = 0.962, TLI = 0.955, SRMR = 0.043), with both convergent and discriminant validity meeting psychometric standards, thereby providing a solid foundation for the subsequent structural model.

To test the chain mediation hypothesis, a path analysis model was constructed using observed variable scores (composite means) derived from the four scales, with wearable device feedback perception as the exogenous predictor, training load management behavior as the outcome variable, and physiological data literacy and recovery self-regulation as proximal and distal mediators, respectively. Composite mean scores were used because the primary analytical objective was to estimate scale-level direct and indirect associations and to maintain consistency with the regression-based bootstrap mediation analyses reported in Tables 1, 2, after the measurement structure of the four constructs had been examined by CFA. However, treating composite scores as observed variables does not explicitly model measurement error, and the resulting path estimates may therefore be influenced by unreliability in the scale scores. The model was estimated using maximum likelihood (ML) estimation, which is compatible with nonparametric bootstrap resampling. A bias-corrected percentile bootstrap procedure (Bollen and Stine, 1990; Shrout and Bolger, 2002) was applied, drawing 5,000 random resamples from the original data to generate indirect effects and their 95% confidence intervals. Significance was determined by whether the confidence interval included zero. The total indirect effect was decomposed into three specific indirect pathways: Path 1—“WFP → PDL → TLM,” Path 2—“WFP → RSR → TLM,” and Path 3—“WFP → PDL → RSR → TLM.” Model evaluation focused on the magnitude and statistical significance of the standardized path coefficients, explained variance (R^2^), and bootstrap confidence intervals for the specific indirect effects. Finally, to rule out confounding effects, age, sex, athletic level, and years of training experience were included as control variables, and path coefficients were estimated after controlling for their influence. All statistical tests were two-tailed, with the significance level set at *p* < 0.05.

**Table 1:** Hierarchical regression results for the chain mediation model (*N* = 357).

Path	Outcome variable	Predictor	β	SE	t	*p*	R^2^
a1	PDL	WFP	0.499	0.048	10.874	< 0.001	0.259
a2	RSR	WFP	0.257	0.053	5.041	< 0.001	0.324
a3	RSR	PDL	0.393	0.051	7.639	< 0.001	
b1	TLM	PDL	0.166	0.052	3.320	< 0.001	0.406
b2	TLM	RSR	0.331	0.052	6.535	< 0.001	0.406
c	TLM (Total)	WFP	0.511	0.049	11.191	< 0.001	0.267
c’	TLM (Direct)	WFP	0.278	0.053	5.622	< 0.001	0.406

β, standardized regression coefficient; SE, standard error. All models control for sex, age, athletic level, and years of training experience. Paths a2 and a3 are estimated from the same regression model in which WFP and PDL simultaneously predict RSR. Paths b1, b2, and c′ are estimated from the same regression model in which WFP, PDL, and RSR simultaneously predict TLM.

**Table 2:** Bootstrap test results for the chain mediation model (*N* = 357, Bootstrap = 5,000).

Effect pathway	Effect	Boot SE	95% CI Lower	95% CI Upper	Significant
Path 1: WFP → PDL → TLM	0.083	0.027	0.032	0.136	Yes
Path 2: WFP → RSR → TLM	0.085	0.020	0.048	0.128	Yes
Path 3: WFP → PDL → RSR → TLM	0.065	0.015	0.040	0.098	Yes
Total indirect effect	0.233	0.031	0.176	0.298	Yes
Direct effect: WFP → TLM	0.278	0.049	0.181	0.375	Yes

Effect sizes are standardized indirect effects. Boot SE, bootstrap standard error; 95% CI, bias-corrected percentile confidence interval. An effect is considered significant (Yes) when the 95% CI does not include zero. WFP, Wearable Device Feedback Perception; PDL, Physiological Data Literacy; RSR, Recovery Self-Regulation; TLM, Training Load Management Behavior.

## 3 Results

### 3.1 Common method bias assessment

Harman’s single-factor test was used as a descriptive diagnostic of potential common method variance. An unrotated exploratory factor analysis of all 42 items extracted four factors with eigenvalues greater than 1, and the first factor accounted for 43.46% of the total variance. However, Harman’s single-factor test has recognized limitations and cannot establish the absence of common method variance (Podsakoff et al., 2003). Given that all focal variables were obtained from the same respondents at a single measurement occasion, common method variance cannot be excluded and may have contributed to the magnitude of the observed associations.

### 3.2 Descriptive statistics and correlations

Table 3 presents the descriptive statistics and Pearson product–moment correlation matrix for all core variables. The mean score for WFP was 3.58 (SD = 0.68), for PDL was 3.47 (SD = 0.71), for RSR was 3.56 (SD = 0.70), and for TLM was 3.56 (SD = 0.73). The absolute values of skewness for all variables were below 1.0, and the absolute values of kurtosis were below 1.5, indicating approximately normal distributions that satisfy the assumptions of parametric testing. Correlation analysis revealed that all four core variables were significantly and positively intercorrelated (all *p* < 0.001). Specifically, WFP showed moderate positive correlations with PDL (r = 0.492), RSR (r = 0.447), and TLM (r = 0.509); PDL exhibited moderate positive correlations with RSR (r = 0.518) and TLM (r = 0.481); and RSR was moderately and positively correlated with TLM (r = 0.542). These significant inter-variable correlations provide preliminary support for the construction of the chain mediation model.

**Table 3:** Descriptive statistics and correlations among study variables (*N* = 357).

Variable	M	SD	Skewness	Kurtosis	1	2	3	4
1. WFP	3.58	0.68	−0.039	−0.342	—			
2. PDL	3.47	0.71	−0.021	−0.357	0.492***	—		
3. RSR	3.56	0.70	−0.215	−0.332	0.447***	0.518***	—	
4. TLM	3.56	0.73	−0.191	−0.437	0.509***	0.481***	0.542***	—

WFP, Wearable Device Feedback Perception; PDL, Physiological Data Literacy; RSR, Recovery Self-Regulation; TLM, Training Load Management Behavior. M, mean; SD, standard deviation.

***p < 0.001.

### 3.3 Chain mediation model testing

To examine the chain mediating effects of wearable device feedback perception on training load management behavior through physiological data literacy and recovery self-regulation, hierarchical regression analysis and bootstrap resampling tests were conducted sequentially, with age, sex, athletic level, and years of training experience controlled.

Table 1 presents the hierarchical regression results for the chain mediation model. Path a1 (WFP → PDL): *β* = 0.499, SE = 0.048, t = 10.874, *p* < 0.001, R^2^ = 0.259, indicating that WFP was significantly and positively associated with PDL. In the model simultaneously including WFP and PDL as predictors of RSR (R^2^ = 0.324), PDL was significantly and positively associated with RSR (Path a3: β = 0.393, SE = 0.051, t = 7.639, *p* < 0.001), and WFP was also significantly associated with RSR (Path a2: β = 0.257, SE = 0.053, t = 5.041, *p* < 0.001). Both PDL (Path b1: β = 0.166, SE = 0.052, t = 3.320, *p* < 0.001) and RSR (Path b2: β = 0.331, SE = 0.052, t = 6.535, *p* < 0.001) were significantly and positively associated with TLM (R^2^ = 0.406). After including both mediators, the direct effect of WFP on TLM remained significant (Path c’: β = 0.278, SE = 0.053, t = 5.622, *p* < 0.001), indicating that PDL and RSR partially mediated the total effect (Total effect: β = 0.511, SE = 0.049, t = 11.191, *p* < 0.001).

Table 2 presents the results of the mediation effect tests based on the bias-corrected percentile bootstrap method (5,000 resamples). The 95% confidence intervals for all three indirect effect pathways excluded zero, as follows: Path 1 (WFP → PDL → TLM): indirect effect = 0.083, 95% CI [0.032, 0.136]; Path 2 (WFP → RSR → TLM): indirect effect = 0.085, 95% CI [0.048, 0.128]; Path 3 (WFP → PDL → RSR → TLM): indirect effect = 0.065, 95% CI [0.040, 0.098]. The total indirect effect was 0.233 (95% CI [0.176, 0.298]), and the direct effect was 0.278 (95% CI [0.181, 0.375]), also with a confidence interval excluding zero. These results indicate a statistically significant pattern of indirect associations consistent with partial mediation within the theoretically specified model.

To evaluate the specified mediation model while controlling for demographic variables, composite scores of WFP, PDL, RSR, and TLM were entered as observed variables in the path model. Because the specified recursive model was saturated, global fit indices were not used to evaluate model adequacy. Interpretation therefore focused on the standardized path coefficients, explained variance, and bootstrap confidence intervals for the indirect associations. The direct association between WFP and TLM remained significant after inclusion of PDL and RSR (β = 0.278, *p* < 0.001), a pattern consistent with partial mediation within the specified model.

### 3.4 Alternative model comparisons

To examine the hypothesized sequential model in relation to plausible alternative specifications, three alternative models were tested. Model A specified PDL and RSR as parallel (rather than sequential) mediators of the WFP–TLM relationship. Model B specified a reversed chain (WFP → RSR → PDL → TLM). Model C dropped the chain pathway entirely. Model comparison relied on two criteria. First, the hypothesized chain model yielded AIC = 613.18 and BIC = 644.20. The parallel mediation model (Model A) yielded AIC = 615.42 and BIC = 646.44, and the chain-dropped model (Model C) yielded AIC = 622.07 and BIC = 653.09; the hypothesized chain model yielded slightly lower AIC and BIC values than the parallel model and lower values than the chain-dropped model. The reverse-chain model (Model B) yielded the same AIC and BIC values as the hypothesized model (AIC = 613.18, BIC = 644.20). Descriptively, the standardized sequential indirect effect was larger for the hypothesized ordering (0.065, 95% BC CI [0.040, 0.098]) than for the reverse ordering (0.028, 95% BC CI [0.011, 0.049]). However, the forward and reverse-chain models were statistically indistinguishable in terms of AIC and BIC. Therefore, the hypothesized ordering was retained primarily on theoretical grounds, and the difference in indirect-effect magnitude should not be interpreted as evidence of temporal precedence or causal direction because the data were cross-sectional.

### 3.5 Diagnostic statistics for the structural model

To assess the adequacy of the maximum-likelihood path estimates, multicollinearity, residual diagnostics, and influential-case analyses were performed on the final model.

Multicollinearity. Variance Inflation Factor (VIF) and tolerance values for all predictors in the final structural model were as follows: WFP VIF = 1.442 (tolerance = 0.693); PDL VIF = 1.574 (tolerance = 0.636); RSR VIF = 1.478 (tolerance = 0.676); covariates VIF range 1.012–1.033 (tolerance 0.968–0.988). All VIFs were well below the conservative threshold of 5, indicating that multicollinearity does not distort the path estimates.

Residual diagnostics. Residuals from the final model exhibited skewness = −0.06 and kurtosis = −0.15, consistent with approximate normality (D’Agostino K^2^ = 0.42, *p* = 0.811). The Breusch–Pagan test indicated homoscedasticity (LM = 6.08, *p* = 0.530). The Durbin–Watson statistic was 2.053, indicating no substantive autocorrelation.

Influential-case diagnostics. Cook’s D values ranged from 0.000 to 0.029 (mean = 0.003); 19 cases (5.3%) exceeded the 4/n heuristic. Standardized residuals ranged from −2.86 to 2.64, with no case exceeding |3|. Leverage values ranged from 0.005 to 0.067; 10 cases (2.8%) exceeded 2(p + 1)/n. No individual case disproportionately influenced the path estimates.

Bootstrap robustness. Indirect effects were estimated using 5,000 bias-corrected bootstrap resamples. Path 1 (WFP → PDL → TLM) = 0.083, 95% BC CI [0.032, 0.136]; Path 2 (WFP → RSR → TLM) = 0.085, 95% BC CI [0.048, 0.128]; Path 3 (WFP → PDL → RSR → TLM) = 0.065, 95% BC CI [0.040, 0.098]; total indirect = 0.233, 95% BC CI [0.176, 0.298]; direct effect c’ = 0.278, 95% BC CI [0.181, 0.375]; these values are consistent with those reported in Table 2. All confidence intervals excluded zero, supporting the stability of the indirect-effect estimates.

## 4 Discussion

### 4.1 The relationship between wearable device feedback perception and training load management behavior

Drawing on a sample of 357 athletes and employing a chain mediation model, the present study examined the associations through which wearable device feedback perception (WFP) relates to training load management behavior (TLM). The positive WFP–TLM association is broadly consistent with previous literature emphasizing the practical value of athlete-reported information in monitoring training responses (Saw et al., 2016). The present study extends this literature by showing that the observed WFP–TLM association was also statistically linked with cognitive (PDL) and self-regulatory (RSR) pathways. Notably, the direct association remained significant (β = 0.278, *p* < 0.001) after including both mediators, indicating that WFP retained an association with TLM that was not accounted for by PDL and RSR.

Correlation analyses revealed that all four core variables were significantly and positively intercorrelated, indicating close covariation among WFP, PDL, RSR, and TLM. The moderate positive correlations indicate that the four constructs covary in the expected directions and are consistent with the hypothesized mediation framework, but also empirically echo the consensus in the training load monitoring literature that multiple monitoring modalities (internal/external load indicators) are interrelated (Bourdon et al., 2017; Impellizzeri et al., 2019; McLaren et al., 2018). Notably, the observed correlations fell within the moderate range, suggesting that while the four constructs are interrelated, they possess distinguishable independence—a finding corroborated by the CFA results showing that the four-factor model significantly outperformed the single-factor model.

### 4.2 The mediating role of physiological data literacy

Path analysis showed that WFP was positively associated with PDL, and PDL, in turn, was positively associated with TLM. The bootstrap test indicated that the 95% CI for Indirect Effect 1 (WFP → PDL → TLM) was [0.032, 0.136], excluding zero, thereby supporting the mediating role of PDL in the WFP–TLM relationship.

An important theoretical contribution of this finding lies in extending the concept of data literacy from the educational domain (Mandinach and Gummer, 2016) to sport, where it specifically captures the interpretation of physiological indicators. Physiological data literacy is defined as the athlete’s capacity to access, interpret, evaluate, and apply physiological data generated by wearable devices for training-related decisions. It differs from health literacy (Nutbeam, 2008), which targets general health information in everyday contexts; from digital and digital-health literacy, which encompass broader competencies for processing and using information in digital health environments (Ban et al., 2024); from technology acceptance frameworks (Venkatesh et al., 2003), which concern the determinants of technology adoption and use; and from self-monitoring (Carver and Scheier, 1998), which describes a self-regulatory process without specifying the data-comprehension component. PDL is therefore best characterized as a content-specific data literacy applied to physiological indicators in sport. This conceptual delineation fills a gap that prior wearable-technology research has not addressed: existing studies (Düking et al., 2016; Seshadri et al., 2019) have focused on the technical validity of devices but have not theorized the cognitive interpretation step.

From a practical standpoint, this finding suggests that sport science practitioners and coaches should recognize that merely equipping athletes with wearable devices is insufficient; complementary systematic data literacy education is needed to help athletes understand the meaning of various physiological indicators, identify anomalous data patterns, and proactively adjust training behavior on the basis of data feedback. This resonates with the survey findings of Akenhead and Nassis (2016), who observed that although most high-level football clubs had extensively adopted training load monitoring technology, a notable practice gap remained in the interpretation and feedback phases. The present study offers a potential explanatory pathway for this gap from the perspective of individual differences among athletes.

### 4.3 The mediating role of recovery self-regulation

The second key finding of this study is the mediating role of RSR in the WFP–TLM relationship. Specifically, WFP was significantly and positively associated with RSR, and RSR was significantly and positively associated with TLM. These results are highly consistent with the cybernetic model of self-regulation (Carver and Scheier, 1982, 1998), which posits that individuals achieve effective self-regulation by comparing their current state with desired goals and adjusting behavior in response to perceived discrepancies. In the context of the present study, wearable devices provide athletes with real-time feedback regarding their physiological states, which may support the identification of recovery needs and the adoption of recovery-related strategies. In the present data, these processes were associated with training load management behavior.

Recent research on athlete self-tracking further suggests that smartphone- and wearable-based monitoring may provide a practical basis for recovery self-management, while also emphasizing the importance of how athletes engage with and act upon the information provided by these technologies (Jakowski, 2022).

The mediating role of RSR also broadens the theoretical scope of athlete recovery research. Prior work has largely focused on the physiological determinants of recovery (Halson, 2014; Kellmann et al., 2018); the present findings indicate that recovery self-regulation may represent a behavioral process linking data-derived feedback with more consistent behavioral routines. These findings are consistent with an extension of Halson’s (2014) monitoring framework in which self-regulatory capacity may help link monitoring information with behavioral responses, rather than implying that monitoring information automatically produces behavioral change.

### 4.4 The chain mediation pathway: the sequential role of physiological data literacy and recovery self-regulation

A key theoretical contribution of the present study lies in proposing and empirically examining the sequential pathway WFP → PDL → RSR → TLM. The significant sequential indirect effect is consistent with a theoretical account in which wearable feedback perception is associated with athletes’ capacity to interpret physiological signals (PDL), which is in turn associated with recovery self-regulation (RSR) and training load management behavior. This proposed ordering is theoretically consistent with self-regulation perspectives in which information processing can inform subsequent regulatory responses (Bandura, 1991). However, the reverse ordering (RSR preceding PDL) remains statistically plausible. Because the data are cross-sectional, the present findings cannot establish that PDL temporally precedes RSR. The WFP → PDL → RSR → TLM pathway should therefore be interpreted as a theoretically proposed sequence that is statistically compatible with the observed data rather than as an established temporal or causal mechanism.

The theoretical value of the proposed pathway lies in integrating data perception, cognitive interpretation, self-regulation, and behavior within a single conceptual framework. In sport training practice, this framework carries several implications. First, wearable-device feedback must be interpretable and meaningful to athletes—a notion consistent with the TAM constructs of perceived usefulness and perceived ease of use (Davis, 1989). When athletes perceive wearable information as useful and understandable, it may serve as a cognitive resource for training-related decision making (Venkatesh et al., 2003). Second, greater cognitive resources may be associated with a transition from passive data reception toward more active use of data in recovery management (Carver and Scheier, 1998). More effective recovery management may, in turn, be associated with a more adaptive balance between training demands and recovery, which is theoretically relevant to the prevention of maladaptive training responses and to effective training load management (Meeusen et al., 2013).

Notably, although the sequential indirect association was statistically significant, the direct association between WFP and TLM also remained significant, indicating that the indirect associations through PDL and RSR accounted for only part of the overall WFP–TLM association. Other variables, such as motivational factors (Hagger, 2010), self-control resources (Baumeister and Vohs, 2007), and self-efficacy (Bandura, 1991), may also be relevant to the observed WFP–TLM association and warrant examination in future research.

### 4.5 Practical implications

The present findings suggest several potential directions for future applied research, although these strategies were not experimentally evaluated in the current study. For coaches, structured data-debriefing sessions may represent one possible approach for helping athletes reflect on HRV, heart-rate-recovery, and sleep-trend information and develop physiological data literacy. For sport psychologists, cognitive-behavioral or guided-reflection interventions could be explored as potential methods for strengthening athletes’ data-interpretation skills and recovery planning. For wearable-device manufacturers, future intervention and usability studies could examine whether trend-based dashboards, annotated physiological thresholds, and brief educational prompts facilitate more accurate interpretation of wearable feedback. Similarly, AI-generated recovery or readiness scores could potentially be more useful when accompanied by educational support that helps athletes understand the physiological basis and uncertainty of algorithmic recommendations. These approaches should be regarded as testable implementation strategies rather than empirically established recommendations derived from the present observational study.

From a load-management perspective, the present study offers a potential lens for interpreting Gabbett’s (2016) “training–injury prevention paradox.” Gabbett emphasized the need to balance “training smarter” and “training harder,” and the present findings suggest that athletes’ ability to interpret wearable-device feedback and regulate recovery may be relevant to the broader concept of “smarter” load management. This finding also resonates with the workload–injury etiology model (Windt and Gabbett, 2017), which underscores the critical role of athlete-level individual factors. Malone et al. (2017) further demonstrated that high chronic training loads, combined with exposure to high-intensity running, reduce injury risk in elite athletes, supporting the necessity of programmed load management. The physiological–biomechanical dual-pathway framework proposed by Vanrenterghem et al. (2017) also provides theoretical guidance for load monitoring.

### 4.6 Limitations and future directions

Several limitations of the present study warrant consideration. First, although this study focused on wearable technologies, the analyses did not incorporate objective device-derived indicators, such as heart rate, heart rate variability (HRV), GPS-derived external load, or device-recorded sleep duration. The findings therefore reflect athletes’ subjective perceptions of wearable feedback rather than the physiological and behavioral data streams generated directly by wearable devices. Future studies should integrate objective indicators, such as device-recorded HRV rMSSD, training load, and sleep duration, with athlete self-reports and coach evaluations to triangulate the proposed feedback-to-behavior pathway.

Second, participants were recruited through convenience sampling from five sport universities and four provincial sport teams in China. This sampling approach limits the generalizability of the findings to recreational athletes, international elite athletes, athletes from other cultural contexts, and younger or older populations. The relatively small numbers of athletes in some competitive-level categories also prevented robust subgroup comparisons. Future research should recruit larger and more diverse samples and use multi-group analysis to examine whether the proposed mediation model is invariant across sport disciplines, competitive levels, age groups, and levels of training experience.

Third, the cross-sectional design precludes causal or temporal conclusions regarding the proposed sequential pathway from PDL to RSR and subsequently to TLM. Although theoretically plausible alternative models were compared, such statistical comparisons cannot establish temporal ordering or causal direction using cross-sectional data. Longitudinal and experimental studies are therefore needed to determine whether changes in wearable feedback perception precede improvements in physiological data literacy and recovery self-regulation and whether these changes subsequently lead to more effective training load management. Future studies could also examine potential moderators, including trust in wearable data, coach support, and team culture.

Fourth, all focal variables were measured using self-report questionnaires collected from the same respondents at a single time point. Although procedural remedies were implemented and the four-factor measurement model fitted the data better than the single-factor model, common method variance cannot be excluded and may have contributed to the magnitude of the observed associations. Future research should use multi-source and temporally separated measurement designs that combine athlete reports, coach assessments, behavioral records, and objective wearable-device data.

Fifth, the measures of PDL, RSR, and TLM remain preliminary and require further independent validation. In particular, the Physiological Data Literacy Scale was adapted from Mandinach and Gummer’s (2016) educational data literacy framework. Although the scale demonstrated strong internal consistency and favorable psychometric properties in the present sample, its measurement invariance across broader populations, test–retest reliability, and applicability across sport disciplines and cultural contexts have not yet been established; although preliminary criterion-related validity evidence was obtained in the present sample (Section 2.2.2), this evidence should be corroborated through independent, cross-sample criterion validation. Further research should comprehensively validate these measures in independent samples and examine their nomological relationships with established sport psychology constructs, including sport confidence, competitive anxiety, and sport motivation.

Sixth, the mediation analyses were conducted using composite mean scores as observed variables, and missing values were handled using single regression imputation. Although the measurement structure of the focal constructs was examined by CFA, the observed-score approach does not explicitly account for measurement error. In addition, single regression imputation does not propagate imputation uncertainty in the same manner as multiple imputation or full-information maximum likelihood. Future studies should examine the robustness of the present findings using latent-variable structural equation modeling and missing-data approaches such as multiple imputation or full-information maximum likelihood.

## 5 Conclusion

Through a cross-sectional survey and path analysis, the present study found that wearable device feedback perception was positively associated with training load management behavior both directly and indirectly through a theoretically proposed sequential pathway involving physiological data literacy and recovery self-regulation. This pathway was statistically compatible with the observed data but should not be interpreted as establishing temporal or causal precedence. The conceptual contribution is to position physiological data literacy as a content-specific form of data literacy that addresses an existing gap in wearable-device research, and to propose a theoretically grounded conceptual framework linking wearable feedback perception, physiological data literacy, recovery self-regulation, and training load management behavior.

## Data Availability

The datasets generated and/or analyzed during the current study are not publicly available due to participant confidentiality and institutional ethics requirements, but de-identified data may be available from the corresponding author upon reasonable request, subject to applicable ethical and data protection requirements.
